# Differences in Perceived and Experienced Stigma Between Problematic Gamblers and Non-gamblers in a General Population Survey

**DOI:** 10.1007/s10899-021-10048-9

**Published:** 2021-07-03

**Authors:** Chiara Andrà, Gabriele Priolo, Francesca Merlin, Claudia Chiavarino

**Affiliations:** 1 Università del Piemonte Orientale, Vercelli, Italy; 2 Istituto Universitario Salesiano Rebaudengo Torino, Torino, Italy

**Keywords:** Dimensions of gambling-related stigma, Psychometric tools, Qualitative and quantitative analysis, Social and self-perceived stigma, Italy

## Abstract

We consider a sample of about 700 people, interviewed on the streets, who are sorted into two groups by a self-report, screening questionnaire: namely, non-problematic gamblers/non-gamblers and problematic gamblers. Within each group, we compare both social (perceived) stigma and self-perceived (experienced) stigma, measured by means of other two self-report questionnaires, and we seek for relations between stigma and socio-demographic variables that can help targeting possible interventions to reduce gambling-related stigma. We, then, compare stigma between the two groups of non-(problematic) gamblers and problematic ones, and we also check the hypothesis that higher social stigma is related to higher self-perceived stigma, as well as higher stigma is related to lesser help-seeking. The latter hypothesis is of utmost importance, given that stigma is recognised to be one of the major causes for hindering help-seeking by problematic gamblers. The research is carried out in Italy, one of the first countries in the world for the money spent *per capita* in gambling activity every year.

## The Importance of Gambling-Related Stigma

Research on gambling-related stigma has increased in the last years, expecially because stigma seems to be the main reason for avoiding treatment by people with gambling problems (Suurvali et al., 2009), or to drop out from treatment before its conclusion (Picucci et al., 2013). It has been shown by Hing et al. (2016) that it is not gambling per se to be stigmatised, namely recreational gambling, but it is problem gambling to be judged, labelled, devalued and discredited. On one hand, our society considers gambling as an “accepted and popular activity” (Quigley et al., 2020, p. 1206), but on the other hand when gambling becomes a disorder it is subject to stigma. Gambling disorder affects a variegated population different for age, sex and social status. It is a phenomenon that, due to the complexity of the factors that compose it, has gradually gone beyond the imaginary closely linked to the Casino: it is now common knowledge that gambling disorder can arise also in public places of common frequentation, including bars and tobacconists, and even at home.

Italy ranks among the first places in the world for legal gambling and this has dramatically increased in recent years: according to a survey conducted by *The Economist*, Italy is among the first five countries in the world for money lost by the population in betting games, with an average annual cost of about 400 euros for each Italian citizen. The spread of gambling in Italy was caused by a progressive deregulation, accompanied by a significant expansion and differentiation of the offer by the gambling sector. This context has inevitably led to situations of addiction by citizens, with estimates suggesting an increase in pathological gamblers of 400% from 2007 to 2017. According to Agipro's (2016) estimate, problem gamblers in Italy would be around one million, with a further 2.5 million of regular users deemed at risk. These numbers do not coincide with the data released by the Italian Ministry of Health in 2015, which counts only 12,376 problem gamblers taken over by the National Health Service. This discrepancy denotes a strong resistance on the part of gamblers to help-seeking for their problematic behavior, and highlights the existence of a large submerged population, also confirming International findings such as Suurvali et al. (2009).

The study on the Italian population by Picucci et al. (2013), which reports 10% of pathological gamblers asking for help, denotes how complex is to accurately quantify the phenomenon and how remarkable is the discrepancy between the number of problematic subjects and the number of requests for help.

In this paper, we investigate the issue of gambling-related stigma by differentiating between the stigma perceived by non-gamblers and the one experienced by problematic gamblers. Among the former group of people, we seek for differences in perceived stigma between those who know problematic gamblers and those who do not. Among the latter group, we investigate help-seeking and reasons for not asking for it. We conduct this research in a country, that is Italy, where gambling is largely diffused and which can represent a relevant case with regard to the interviewees and their relationships with gamblers and with the activity of gambling.

## Those Who Stigmatise and Those Who Are Stigmatized

Historically, Greeks used the term "stigma" to indicate a sign imprinted on the skin of individuals associated with reprehensible aspects considered linked to a negative "moral condition". Stigma were signs attributed to categories such as criminals, slaves or traitors, to identify them as defective people who therefore had to be avoided, particularly in public places (Goffman, 1963).

At present days, as noted by Link and Phelan (2001), there is no univocal definition of stigma: the multidisciplinarity and transversality of the phenomenon have produced multiple contributions from fields of research that are very far from each other. However, it is possible to find some points in common in order to arrive at a transversal definition. Horch and Hodgins (2008) for example to explain the concept of gambling-related stigma borrow the definition by Crocker et al. (1998) of stigma in the field of mental illness. According to this definition, stigma represents the devaluation of a person in a particular social context based on the presence of a social attribute or identity perceived as negative. In his seminal work, Goffman (1963) argues that the management of stigma is present in any society, as moral rules for the definition of identities come into play. The mechanism underlying stigma should, thus, be considered a necessary condition for the existence of any society, as it constitutes a defense mechanism aimed at creating expectations towards the people we come into contact with (Goffman, 1963).

Conversely, the stigmatized subject responds to stigmatization by enacting defensive mechanisms (Goffman, 1963). Among the most common ones, Goffman lists: correcting the aspects underlying the stigmatised’s social isolation, attempting to integrate into the activities from which the stigmatised expects to be excluded by trying to excel at them, using the stigma to obtain advantages, for example claiming for one own's difficulty as an excuse for failure. The fourth defense mechanism identified by Goffman (1963) consists in considering pain as a privilege and this seems to relate in particular to religious contexts, expecially Christianity, where misfortune leads to a reward after death. The last defense mechanism identified by Gofffman (1963) consists in highlighting the limits of the subjects deemed as "normal", thus not stigmatised.

For Goffman (1963) there are also people who support the stigmatised ones, sharing with them the conviction that they are “essentially normal” human beings. The first one is the category of people who share the same stigma with the individual (the group of "peers"), with which one can work to improve one's condition in the eyes of the society through multiple initiatives. The second category is that of "wise men", belonging to the world of non-stigmatised people who, for particular reasons, understand and participate in the private life of stigmatized individuals and are somehow accepted by the group. This leads to the hypothesis that, among the non-stigmatised people, there can be different perceived stigma towards those who are stigmatised, and this may hold true expecially for those who have a relative, or a friend, who possesses the features that lead to stigmatization.

## What Is Stigmatised

Stigma varies not only with respect to people and their perception, but also with respect to its object. Addictions bear higher stigma than other psychiatric pathologies or social conditions. This has been demonstrated by Schomerus et al. (2011), which compared 17 representative studies regarding alcohol addiction stigma related to other psychiatric distress. It was found that addictions are considered as a “sought” risk by the patient compared to other mental pathologies and socially unfavorable conditions (Schomerus et al., 2011).

Carroll et al. (2013) conducted a qualitative study on gambling stigma and asking for help. The research results indicated that gambling problem is not well understood by society and that this contributes to the stigma associated with having a gambling addiction. Society's approaches to problem gambling can be contradictory. For example, people with gambling problems are seen as people with individual personality defects, and gambling disorder is not understood as an addiction in the same way as drug and alcohol addictions. While society has greater empathy for people with drug or alcohol problems because they are recognized as addicts, problem gamblers are more likely to be blamed by society for their addiction (Carroll et al., 2013). This was also demonstrated by Horch and Hodgins (2008), who compared stigma on gambling versus other stigma. The results showed that the most stigmatized condition is gambling addiction. This can lead one to think that the lack of an addictive substance aggravates the idea that the employee is aware of her actions and that, therefore, the abuse of a certain behavior is due more to a sense of irresponsibility than to a disorder. In a follow-up study, Horch and Hodgins (2013) compared the attributes most and least attributed to gamblers: the respondents associated morally negative characteristics to problematic gambling, insinuating with attributes such as "weakness of will" and "irresponsibility" and claiming that it is a vice more than a disease. Horch and Hodgins (2013) provide also evidence that the social stigma is not only present, but that there is a heavy self-perceived stigma on the part of the gamblers themselves. To this regard, Luoma et al. (2007) offer an interesting interpretation: it is the gambler's belief in the presence of stigmatizing thoughts that affects his beliefs and behaviors, regardless of whether these thoughts are concretely exercised or not. Therefore, it is as if the problematic gambler takes on an ancestral cultural baggage rather than simply reacting to a behavior of others perceived concretely.

Hing et al. (2016) also studied the gamblers’ perspective and participants expressed concerns about being seen as "problem gamblers". The consequences more feared are: degrading stereotypes, social rejection, hostile responses and devaluing behaviors. Many participants also internalized perceived stigma as self-stigma, with reported deleterious effects on self-esteem, self-efficacy, perceived social worth, and mental and physical health. Deep shame is an almost universal emotion and exacerbated by a possible relapse. Secrecy appears to be the main coping mechanism used, with self-stigma proving to be the main obstacle to coming out and seeking help (Hing et al., 2016).

What has been studied in depth about stigma, indeed, has been its role in requesting for help. A study by Horch and Hodgins (2015) found out that self-stigma was largely composed of shame and a low sense of self-worth, and the most widely used strategies were withdrawal and secrecy. The authors further observed that the acceptance of the most serious stereotypes towards problem gamblers corresponded to a decrease in the request for treatment. Conversely, among the predictors of a greater request for help Horch and Hodgins (2015) identified: more severe addiction, being male, high income and a positive attitude towards treatment.

Hing et al. (2016) also investigated how stigma affects seeking help, but the peculiarity of the study is that it was not the statements of the gamblers that were analyzed, but the interviews made with 9 consultants for problems related to gambling in Victoria, Australia. The interviews were studied through interpretative phenomenological analysis. Consultants indicated that problem gambling is often compounded by stigma and its effects. Stigma is created and maintained by a lack of public understanding of problem gambling and its causes, and internalized through self-stigmatizing beliefs on the part of the gamblers, leading to delaying seeking help, increasing anxiety, to fear the consultant's opinion and to be afraid of any relapses. Counselors argued that before they began effective gambling behavior treatment, they needed to help clients overcome their stigmatizing beliefs to build trust and alliance, restore self-esteem, improve abilities to suppress stigma and to foster the belief that recovery was possible. Support from other people significant to the gambler and preparing clients for relapse are also important elements in lowering the stigma. Hing et al. (2016) conclude the study by stating that addressing stigma at the start of treatment can help improve adherence and recovery during the surgery.

A study by Cunningham et al. (2009) also included stigma among the three causes of failure to help seeking. According to this study, the request for help would be mainly undermined by the conviction of being able to solve the problem with one's own strength, minimizing the nature of the problem.

Suurvali et al. (2009) reviewed the empirical studies concerning the resistance to seeking help for gamblers, taking into account documents and reports in English published since 1998. 19 studies conducted in five countries were identified. Despite differences in methodology, the same barriers to treatment were often identified and the most commonly reported ones were: the desire to manage the problem alone; stigma and shame; the reluctance to admit the problem; contact with problems regarding the treatment itself. The research authors argue that reluctance to admit the problem may be even more prevalent than evidenced by the results. Other frequently reported barriers include lack of knowledge about treatment options and practical issues related to treatment frequency.

Regarding resistance to seeking help, particularly when referring to the belief that one can do it alone or to the presence of a strong stigma, Carroll et al. (2013) affirm the need to encourage the public to view seeking help for gambling problems as a courageous and responsible action, rather than a sign of weakness or despair, leading gamblers to view seeking help not as a surrender but as a strength.

However, it is also important to consider how stigma and request for help are linked to one's own cultural background. A recent study by Radermacher et al. (2016) is illuminating in this regard. It aimed at investigating the perception of gambling in two ethnic minorities (Tamil and Chinese) in Australia, correlating it with “saving face” and resistance in asking for help. It emerged that the Chinese consider gambling as a natural element of life, “part of their blood” (Radermacher et al., 2016), until it becomes pathological. For the Tamils, on the other hand, gambling is considered a “sin” in itself. In the Chinese population one is ostracized only if gambling behavior becomes pathological, while in the Tamil population one is stigmatised as a gambler. The study is interesting because it highlights how different radicalized cultures in a multiethnic context can perceive social problems related to addictions differently, even if the two groups converge in considering the request for help from professionals a stigmatizing factor to be avoided and to be undertaken only in the onset of a serious acute crisis, preferring instead to solve the problems in the intimate family environment. For both populations, therefore, the social weight of addiction turns out to be once again a barrier to "help-seeking", as well as confirming the synchronic relativity of the phenomenon in relation to the reference society. This study on the socio-psychological perspective of stigma at the local level does not find analogous examples in the Italian scientific literature, which seems focused on mapping the phenomenon of gambling itself.

It would therefore be desirable to conduct studies capable of identifying the problems linked to the failure to request assistance with local initiatives taking into account the social, economic and cultural contexts.

## Perceived Versus Experienced Stigma

In our view, research on stigma has at the present two main limitations. The first one is that the majority of studies relies on a sample population of university students and thus leading to possible biases on perceived stigma (Hing et al., 2016). A second limitation is that a few studies have used psychometric tests: more or less structured interviews are often used and the studies are mainly qualitative. This is because the development of appropriate measurement scales is very recent and their use to date is relatively rare for gambling-related stigma, although there are many existing tools aimed at measuring stigma with regard to subjects with mental illnesses and substance abuse.

Therefore, the development, validation and use of scales are to be considered a critical step in the analysis and study of stigma.

Fundamental in this regard is the work done by Donaldson et al. (2015), which led for the first time to the development of two tools capable of measuring the level o﻿f both perceived and experienced stigma, or social and self-perceived ones respectively.^1^ In particular, the Gambling Perceived Stigma Scale (GPSS) is aimed at measuring the perception of stigma at social level by the general population (Donaldson et al., 2015), while the Gambling Experienced Stigma Scale (GESS) is aimed at measuring the stigma perceived by gamblers themselves. Their research took place in Australia, where problem gamblers are estimated to be 1% of the population, with a further range from 1.4 to 2.1% considered at risk of DGA (Productivity Commission, 2010). Among problem gamblers in Australia, according to Productivity Commission (2010), only 8–17% ask for help and this is much less than other mental illnesses, whose frequency of request for help stands at around 46% (Whiteford et al., 2014). Underlining how stigma is one of the main barriers to seeking help, Donaldson et al. (2015) identify the need to validate a specific tool in order to measure the amount of social and self-perceived stigma. In fact, there are no similar previously validated tools, and previous studies have always been carried out using tools to measure the stigma for other mental pathologies and disorders related to substance use (Donaldson et al., 2015).

The two scales GPSS and GESS have been independently validated (Donaldson et al., 2015).

For GPSS, to select the most appropriate items, the authors referred to the multiaxial classification made by Jones (1984), who identified six universal categories according to which stigma is described in the scientific literature: concealment, course, pervasiveness, aesthetic quality, origin and danger. The dimension of concealment concerns how clear the condition or behavior is for others and to what extent this visibility can be controlled by the addicted individual. Problematic behaviors can be easily hidden, more than other types of addiction (Horch & Hodgins, 2008). However, this has been recently criticised by Hing et al. (2016), who found out that a large percentage of the people interviewed for their study believe that problematic gambling is somewhat noticeable for family and friends, as if there are “signs of problem gambling” (p. 859), which can be recognised by the gambler’s significant others who can respond to the problem in time.

The dimension of course relates to the perceived possibility of change. For example, the stigma associated with mental illness often refers to the belief that the condition is permanent (Björkman et al., 2007). This dimension is relevant to gambling, since it is common opinion that all gamblers are destined to become problem gamblers, and that once one becomes a gambler she will be a gambler forever. Misconceptions regarding the persistence of addiction are also of particular importance in understanding the beliefs that can hinder seeking help and treatment (Horch, 2011; Suurvali et al., 2009).

The dimension of pervasiveness refers to the extent to which the condition or behavior can hinder interpersonal interaction and communication. This is of particular importance for gambling, which requires the investment of time subtracted to other activities, which is identified as a fundamental cause of harm experienced by family and friends (Darbyshire et al., 2001; Dickson-Swift et al., 2005).

Aesthetic qualities reflect the extent to which the person with the stigmatized condition or behavior becomes less attractive or more repulsive as a function of that condition or behavior. For example, the physical attributes associated with obesity can lead to perceptions of laziness, poor hygiene and lack of attractiveness (Lillis et al., 2010), while the stigma associated with homelessness relies heavily on the aesthetic dimension due to the difficulties associated with accessing resources to participate in cleaning and grooming (Phelan et al., 1997). In relation to the stigma of gambling, it has been hypothesized that the aesthetic dimension may focus more on character (perception of greed, laziness or irresponsibility) than on physical appearance (Donaldson et al., 2015).

The dimension of origin reflects perceptions about the circumstances in which the condition or behavior arose, including the attributions of related responsibilities. Research has found that attributions of individual responsibility are often associated to HIV (Kalichman et al., 2009; Rutledge et al., 2011) and cancer lungs (Cataldo et al., 2011), as well as to alcoholism (Peluso & Blay, 2008) and miscarriage (Kumara et al., 2009). This dimension has a strong influence on the internalization of responsibilities and can be a barrier both for seeking help and for offering support (Derlega et al., 2010; Horch, 2011; Kalichman et al., 2009; Rutledge et al., 2011). The attribution of origin in relation to gambling relates to how it began and progressed, as well as the degree to which responsibility for gambling problems can be attributed to individual factors (e.g., character weakness) or situational factors (e.g., role models or the need to escape stressful life events). Attribution theory suggests that when an individual's condition or behavior is considered to be under the individual's control, or if the individual is responsible for it, others are more likely to deny help and avoid the person (Corrigan et al., 2003; Weiner et al., 1988). These behavioral responses are related to feelings of anger towards the individual concerned. Conversely, if the condition is considered to be due to situational rather than individual factors, then there may be feelings of pity from others, who can offer help (Corrigan et al., 2001).

The dimension of risk reflects the perceived likelihood, imminence or severity of the risk to others. Danger to others is commonly associated with incarceration (LeBel, 2012), mental illness (Björkman et al., 2007), alcoholism and substance abuse (Peluso & Blay, 2008; Room, 2005; Schomerus et al., 2011). While the perceived risk or danger in relation to violence or physical harm may not be relevant to gambling (Afifi et al., 2010), stigma associates with related gambling behaviors hypotheses of criminal behavior, financial risk, impact on family well-being or dishonest behavior (Donaldson et al., 2015).

Starting from this theoretical framework, Donaldson et al. (2015) identified 90 studies in the scientific literature concerning scales for gambling-related stigma and for other stigma, provided that the former can have similar features of, and thus fit within, the latter. Starting from the eight scales thus identified, 150 items were selected and divided between social stigma and self-perceived stigma. By excluding repetitions and selecting for relevance, the authors maintained a final group of 36 items relevant to social stigma of gambling and other 18 items relevant to self-perceived stigma. These were then validated in a large population sample of 1370 Australian adults.

In the calibration of the GPSS, the authors maintained 13 items in total. They also identified two subscales of stigma, referable to two behavioral tendencies of the stigmatizing subjects, namely: contempt, characterized by expressions such as "most people think that gamblers are liars", and ostracism, characterized by expressions such as "most people would avoid contact with a gambler". The historical reference to ostracism, which dates back to the sixth century B.C. in Athens, chosen by Donaldson et al. (2015), carries both a political and a moral meaning and highlights how the phenomenon of stigma has not only the function of marking a behavior as deplorable, but also a saving vocation on the part of those who exercise it, which sees the stigmatized as a potential social danger to be isolated in order to preserve the health of the whole society. With reference to both subscales, although the factorial structure analysis did not support the six-dimensional stigma structure adopted by Jones et al. (1984), the identified dimensions of contempt and ostracism align with this framework, particularly in the areas of aesthetics and risk (Donaldson et al., 2015). Thus, even if a six-dimensional stigma model does not appear to be the most efficient framework within which to assess gambling-related stigma, maintaining this theoretical frame of reference is relevant to understand the origin of gambling-related stigma scales (Donaldson et al., 2015).

As regards the items related to GESS, the authors report a greater simplicity on the one hand, only the sample of gamblers being investigated, but greater difficulty on the other hand, since less data is available (Donaldson et al., 2015). The authors opted to identify a one-dimensional test. This decision was made considering the fact that the studies deemed most reliable by Donaldson et al. (2015) rejected the hypothesis of multiple dimensions. The study led the GESS to be composed of 13 items.

To date, these two questionnaires remain the only existing ones that have been validated to psychometrically measure the amount of social and self-perceived stigma in gambling, offering a useful tool for investigating the phenomenon.

## Hypotheses of Research

The hypotheses of our research are four. The first one aims at finding possible relations between the perceived stigma (GPSS) and socio-demographic variables like gender, number of people living with, occupation, income and education. Previous studies on other stigmatized diseases, including HIV, provide evidence that stigma is influenced by multiple socio-demographic variables (Logie & Gadalla, 2008) and Donaldson et al. (2015) show that men are more inclined to gamble, while women are more stigmatizing. If results by Donaldson et al. (2015) are confirmed in our study, and if the one by Logie and Gadalla (2008) can be extended to the case of problematic gambling, our study would contribute to the understanding of the role of various socio-demographic variables in stigmatisation, and hence to reflect on which ones it could be possible to intervene in order to reduce stigma, but also to target the interventions in favor of stigma reduction.

The second hypothesis is that there is a difference, both quantitative and qualitative, in the perceived stigma between those who gamble and those who do not. Based on research by Donaldson et al. (2015), it is hypothesized that the stigma perceived by non-gamblers is higher than that of gamblers and, according to the research by Horch and Hodgins (2013), that the stigma by non-gamblers is more about judgments of moral value than the gamblers. In addition, non-gamblers will be divided between those who know gamblers and those who have no knowledge of people who gamble. Based on some studies carried out on heavily stigmatized diseases such as HIV (Prati et al., 2016), the hypothesis is that those who know gamblers have a lower perceived level of stigma than those who do not.

The third hypothesis is that a high level of perceived stigma leads to an increase in the self-perceived stigma on gamblers, and that these two factors affect the seeking for help. This hypothesis is based on the data found in the literature that failure to requesting help is often caused by high social and self-perceived stigma, which causes feelings of shame and lack of self-efficacy (Hing et al., 2016; Horch & Hodgins, 2015; Suurvali et al., 2009). It is therefore hypothesized that, among the gamblers, those who asked for help differ from those who did not, due to a higher level of social and self-perceived stigma.

The fourth hypothesis aims to investigate the correlation between self-perceived stigma and the severity of gambling behavior. This hypothesis is based on the data found in the literature that high stigma influences the cure, the treatment and the course of the disease in heavily stigmatized pathologies including HIV (Rueda et al., 2001).

## Methodology

### Participants

The survey was promoted by IUSTO—Istituto Universitario Salesiano Torino Rebaudengo, supported by the “Giovanni Agnelli” Foundation in Torino, Italy. The area taken into consideration for the study is District 6 of Torino, which is characterized by a high level of social hardship, cultural and economic poverty. Addressing Hing et al.’s (2016) plea for studies based on samples different from university students, we considered a population with features that have the potential to well represent the phenomenon of gambling and of problematic gambling, but also of stigma.

704 valid responses have been collected (55 from 759 raw data had too many missing entries to be considered for the study). Among the respondents, 46.6% declared to be resident in District 6, 54.4% are females, 95% have Italian nationality, 56% are workers, 92% live with others, while 8% live alone. A majority of the interviewees are aged 18–34 (48%), 29.3% are aged 35–54, 17.8% are aged 55–74 and the remaining 3% are older than 74 years. The majority of respondents have a full-time job (41%), while 8% have a part-time job, 7% are occasional workers, 2% are housewives and 24% are students. 11% are retired. With respect to the annual income, 26.9% earn less than 5000 euros, 27.2% earn between 5000 and 15.000 euros, 23.8% between 15.000 and 25.000 euros, 12.5% between 25.000 and 35.000 euros and 9.4% earn more than 35.000 euros annually. Finally, 25.8% of respondents declare to have a middle school diploma or less, 45.4% have a high school diploma, 26.7% have graduated and 2% have a postgraduate degree.

Gamblers represent a percentage of 6.2% of the sample.

In the sub-sample of non-gamblers, 56.3% declare to know at least one person who gambles: for 4.4% of them it is a parent, 4.4% a sibling, 2.7% their partner, 18.7% someone else in the family, 43.6% a friend and 49.8% someone they know but with whom they do not have a particular relationship.

### Tools

The tool used to conduct the survey is a battery of five questionnaires already validated in the International literature and translated in Italian through the back-translation procedure as they lack an official Italian translation at the present. The questionnaires were divided into three sections. The first section, administered to all participants, includes three parts: (a) the Gambling Perceived Stigma Scale (GPSS; Donaldson et al., 2015), composed of 13 items with Likert scale responses ranging from 1 ("strongly disagree") to 4 ("strongly agree"), which, in addition to providing the total score, identifies the two subscales related to ostracism (or isolation, characterized by the evaluation of expressions such as "many people would feel uncomfortable communicating with a gambler”) and contempt (or disgust, characterized by expressions such as “most people think gamblers are liars”). Both total score and subscales have good reliability: for the total score α = 0.87, for contempt α = 0.89, for ostracism α = 0.87 (Donaldson et al., 2015); (b) the 8-item Attitudes Towards Gambling Scale (ATGS-8; Canale et al., 2016), composed of eight items with responses on Likert scale from 1 ("strongly disagree") to 5 ("strongly disagree"), which has good reliability: α = 0.78 (Canale et al., 2016); (c) the Consumption Screening for Problem Gambling (CSPG; Rockloff, 2011), a screening questionnaire with three items with a score from 1 to 4, with a cut-off of 3 points above which one is considered a problem gambler.^2^ The reliability is very high, with α = 0.93 (Rockloff, 2011).

The second section has been administered only to those who have scored more than 3 points in the CSPG and thus can be considered as problematic gamblers. The section opens with a series of questions relating to the gambling activity: where does one gamble, what are one’s favorite gambling games and whether one has ever asked for help with gambling behavior (if so to whom, if not why). Then, two questionnaires are presented: (a) the Gambling Experienced Stigma Scale (GESS; Donaldson et al., 2015), composed of 13 items with responses on Likert scale from 1 ("strongly disagree") to 4 ("strongly agree"), and aimed at measuring self-perceived stigma, with expressions like “I think I should be ashamed of myself for my propensity to play". Validity was measured by the authors with a Tucker-Lewis index of 0.922 (Donaldson et al., 2015); (b) the Problem Gambling Severity Index (PGSI; Currie et al., 2012), composed of 9 items with responses on Likert scale from 0 ("never") to 3 ("almost always"). The test has good reliability, with α = 0.84 (Currie et al., 2012).

The third section is administered to non-problematic gamblers and asks if one knows at least one gambler. In particular, it asks the kind of relationship with the latter and, if it is known, whether there has been a request by this person for help (if so to whom, and if not why). Open-ended questions have been asked, thus qualitative answers have been collected.

### Method of data collection

The questionnaires were administered in the territory of District 6 of Turin, mainly in public places (such as supermarkets and gyms) and on the street.

In particular, the interviewees were asked to fill in a hard copy of the questionnaires, explaining that the goal was to detect people's opinion about gambling, as part of a university research. It was specified that the questionnaire was completely anonymous, and that the data would then be analyzed only in aggregate form.

### Method of data analysis

The completed questionnaires were signed with a progressive alphanumeric code. The data were then organized into a matrix using Microsoft's Excel software. Statistical analyzes were then carried out using IBM's SPSS program.

In order to investigate the first hypothesis, Student’s t-test on the difference between means for binary variables like gender, or ANOVA for multi-categorical variables like education, have been conducted to measure the differences in social stigma (GPSS) and attitude towards gambling (8-ATGS) scores with respect to the socio-demographic features taken into consideration for the study.

To investigate the second hypothesis, a Student’s t-test has been carried out to compare the group of non-(problematic) gamblers with that of problematic gamblers with respect to the GPSS, and subsequently a one-way ANOVA to compare the groups of non-gamblers who do not know gamblers, non-gamblers who know gamblers and problematic gamblers with respect to GPSS and 8-ATGS. To recall, problematic gamblers are distinguished from non-gamblers/non-problematic gamblers on the basis of their responses to the Consumption Screen for Problem Gambling (CSPG), administered in the first section of the questionnaire.

To investigate the third hypothesis, a linear regression was performed between the total score of the GPSS (social stigma, independent variable) and the GESS score (self-perceived stigma, dependent variable) on the subsample of problematic gamblers identified with the CSPG. A further linear regression was performed with the two GPSS subscales (ostracism and contempt) as independent variables (instead of the total score). Subsequently, a Student’s t-test was performed on the differences both in GPSS (social stigma) and in GESS (self-perceived stigma) mean scores between problematic gamblers who asked for help and those who did not. Open answers on the reasons for asking for help or not were also reported.

To investigate the fourth hypothesis, a correlation was made between the scores of the GESS (self-perceived stigma) and those of the PGSI (severity of gambling behavior).

## Results

### Relations between socio-demographic variables and social stigma and attitudes towards gambling

#### Gender

Roughly, as it emerges from the description of the participants to the study, half of the interviewees are males and the other half are females. However, 12% of males are problematic gamblers (namely, they scored 3 or higher in the CSPG), whilst only 1.4% of females are. This confirms the hypothesis that males tend to be problematic gamblers more than females. With respect to the scores for both GPSS (total, contempt and ostracism) and 8-ATGS, females have higher average scores for the former (t = 0.257, t = 0.201 and t = 0.240 respectively) and lower scores for the latter (t = − 0.358) compared to males (t represents the difference between the averages of the scores on the female subsample minus the males one), but these differences are not statistically significant.

#### Living alone

Only 56 (8%) of the respondents live alone and 9.7% of them are problematic gamblers. Among the majority of the respondents who live with someone, 5.4% are problematic gamblers. However, the differences in the scores of GPSS and 8-ATSG are not statistically significantly different. This suggests that to live with someone may alleviate gambling disorder, but has no effect on neither stigma nor attitude with respect to gambling.

#### Occupation

Workers represent approximately 50% of the sample, being the other half composed of full-time students, retired workers, unemployed and housewifes. 7% of non-workers and 5.5% of workers are problematic gamblers: these differences are not statistically significant, as well as the differences in the scores of GPSS and 8-ATSG.

#### Income

371 interviewees (51.4% of the sample) have an annual income up to 15.000 euros. Among them, 7.8% turn out to be problematic gamblers. Among those with an annual income higher than 15.000 euros, only 4.56% are problematic gamblers. Differences in the GPSS and 8-ATGS scores are not significant. This suggests that problematic gambling is more frequent for low-income people, but stigma and attitude are not influenced by the salary.

#### Education

There is a statistically significant difference (p = 0.000) with respect to the educational qualification for gambling disorder, namely the higher the qualification the lower the percentage of problematic gambling. Also attitude towards gambling (8-ATGS) has a statistically significant relation with education, as the higher the qualification the more negative the attitude. Stigma (GPSS) is not statistically different with respect to this variable.

### Differences in stigma between problematic gamblers and non-problematic/non gamblers

To investigate the second hypothesis, a t-test on the mean difference in the scores of the GPSS (social stigma) between the two groups of non-problematic/non gamblers and problematic gamblers. There is a significant difference between the groups both as regards the total score (t = 4.92, p < 0.001; gamblers M = 30.06, non-gamblers M = 35.09), and as regards the GPSS subscales of contempt (t = 3.16, p = 0.002, gamblers M = 14.17, non-gamblers M = 16.14) and ostracism (t = 2.56, p = 0.011, gamblers M = 14.03, non-gamblers M = 15.73). Non-problematic gamblers report higher ostracism and higher contempt, thus higher stigma overall, than problematic gamblers and this confirms previous results in literature.

From an analysis of the individual items of the questionnaire, it emerges that in all items the non-problematic gamblers revealed a higher average score than problematic ones. The largest differences can be seen for items 7 ("most people believe that gamblers have no self-control"), 4 ("most people think that gamblers cannot handle responsibilities"), 3 ("most people think gamblers tend to be untrustworthy") and 2 ("once they know that a person is a gambler, most people will take their opinion less seriously") of the GPSS. This confirms the hypothesis that non-problematic gamblers show higher stigma than those who gamble, and that some traits of stigma create a wider divide between the two groups of people, in particular the issues of self-control, being responsible and trustworthy.

The scores obtained by problematic gamblers and non-problematic/non gamblers in the attitude test for gambling (8-ATGS) were also compared using the t-test and the results revealed a significant score difference the groups (t = 6.10, p < 0.001), with an attitude towards gambling significantly higher for problematic gamblers (M = 22.95) compared to non-problematic ones (M = 17.34).

In order to test the second part of the second hypothesis, a one-way ANOVA was performed to compare perceived stigma (GPSS scores) in three groups: problematic gamblers, non-problematic/non gamblers who don't know problematic gamblers and non-problematic/non gamblers who know gamblers. The results show that there is a significant difference between the three groups (F = 8.7, p < 0.001). Specifically, problematic gamblers show lower scores (M = 30.1) than both non-gamblers who don't know gamblers (M = 35.7) and non-gamblers who know gamblers (M = 34.8, p < 0.001 in both cases). However, the difference between non-gamblers who do not know gamblers and non-gamblers who know gamblers is not statistically significant.

From the descriptive comparison of the individual GPSS items, it emerges that item 6 ("most people think gamblers are greedy") is less rated by the group of non-gamblers who know gamblers than non-gamblers who don’t know problematic gamblers. Overall, it can be observed that, albeit on the feature of greed, non-gamblers who know gamblers are less stigmatizing than non-gamblers who don't know gamblers.

A comparison among the three groups (gamblers, non-gamblers who know gamblers and non-gamblers who don't know gamblers) was also carried out with ANOVA for the scores of the 8-ATGS (attitude towards gambling). The data show that there is a significant difference between the three groups (F = 19.43, p < 0.001). Specifically, gamblers show a higher average (M = 22.95) with respect to non-gamblers who do not know (M = 16.87, p < 0.001) and those who know (M = 17.62, p < 0.001) gamblers. The mean difference between the two groups of non-gamblers, however, is again not statistically significant (p = 0.196). Item-wise, the only relevant difference in terms of averages concerns item 8 ("it would be better if gambling were completely prohibited"), which non-gamblers who do not know gamblers are more in favor of.

### Social and self-perceived stigma and the request for help

A linear regression was performed on the sample of identified problematic gamblers between social stigma (GPSS) as an independent variable and self-perceived stigma (GESS) as the dependent variable. The analysis shows that social stigma significantly predicts self-perceived one (p = 0.008), explaining 18.9% of the variance.

To further investigate the contribution of social stigma to the observed effects on self-perceived stigma (GESS), a further linear regression was performed with the two GPSS subscales (ostracism and contempt) as independent variables and the GESS score as the dependent variable. No significance emerged with regard to the contempt subscale (p = 0.466), while significance emerged with regard to ostracism (p = 0.003).

To evaluate how much the two types of stigma (i.e., social and self-perceived ones) influence the request for help, a t-test was carried out to compare those who asked for help and those who did not on the scores of the GPSS and of the GESS. The difference between the two groups was not significant for the GPSS, while it was significant for the GESS (t = − 2.327, p = 0.026), indicating that gamblers who ask for help live with a significantly lower self-stigma than those who do not ask for it.

A descriptive analysis of requests for help was also carried out, distinguishing the answers provided directly by the group of problematic gamblers and the answers reported by the group of non-gamblers who know problematic gamblers. In the first case, among the gamblers interviewed, only 26.3% declared that they asked for help. Of these, 40% would have done so with relatives and friends, 20% through telephone support and 10% would have instead gone to a support center.

As for the motivation behind the non-request for help, whose response was free and not stratified into selectable options. The conviction of not needing it emerged in a majority, with a percentage of 44%, followed by the conviction of being not problematic (31%). Other responses were: the belief of gambling a few (9%), the claim of spending not so much money (6%), the belief of being able to control one's own gambling activity (6%) and the claim to spend their money in the way they want (3%).

As for the problematic gamblers known by the interviewees (thus, not directly interviewed), it emerged that 15.8% made at least one request for help. 18.5% of the interviewees say they cannot say nothing about it, since they do not know whether help has been asked. On the type of help requested by known problematic gamblers, it emerged that 46.3% asked to relatives, 26.8% to the National Health Service, 12.2% to a support center, 12.2% to self-help groups, 4.9% requested online help and 4.9% consulted a private professional.

On the alleged reasons described regarding the lack of a request for help from known problematic gamblers (which, like the problematic gamblers directly interviewed, were not stratified into selectable options), the most recurrent answers were more varied than the gamblers directly interviewed: 24.4% reported a gambler's lack of awareness of their problem; 16.8% reported that the gambler is not in a problematic condition, mostly playing occasionally; 6.2% reported that a feeling of shame was hidden behind the failure to request help; 3.5% claimed that the known gambler is free to spend their money without having to account for it to anyone.

### Severity of gambling behavior and self-perceived stigma

A Pearson correlation between GESS (self-perceived stigma) and PGSI (severity of gambling behavior) was made to investigate the fourth and last hypothesis on the sample of problematic gamblers. A significant, strong and positive correlation emerged (r = 0.635, p < 0.001), suggesting that greater severity of gambling behavior corresponds to greater self-perceived stigma.

## Discussion

The first aim of our research was to explore differences in social stigma with respect to socio-demographic variables. The results lead us to say that social stigma does not depend on gender, living alone/with someone, being employed, income and education. At a closer look, considering gender, Donaldson et al.’s (2015) results are only partially confirmed: we confirm that males tend to gamble much more than females, being differences statistically significant, and females have higher social stigma and less positive attitude towards gambling, but the latter turns out to be not statistically significant in our findings. Living with someone (i.e., not alone) seems to reduce problem gambling, but has no effect on either social stigma or attitude related to gambling. Being employed or not does not lead to statistically significant differences in gambling, its stigma and attitude towards it, but a lower income is related to higher percentages of problematic gamblers. Social stigma and attitude do not vary with respect to different incomes, indeed. Education turns out to be related to problem gambling and attitude, but not significantly to social stigma: the higher the qualification, the lower the percentages of problematic gamblers with that qualification and the less positive the attitude towards gambling. That education helps to increase negative attitudes towards gambling and developing awareness about risks of addiction has been evidenced by studies like Andrà et al. (2015, 2016). All in all, however, socio-demographic variables help explaining gambling risks and identifying groups of people who can be more exposed to becoming problematic gamblers and school education plays a significant role in developing negative attitudes towards gambling, but social stigma seems to be equally spread across varying income and instruction level, regardless of being employed or not, or living alone or not.

Indeed, a limitation of this study was to categorise socio-demographic variables in a generic way. For example, the educational qualification was asked, regardless of its type. Being employed or not has been recorded, regardless of the specific job. In order to obtain statistically sound results on all these nuances would have requested an even larger sample, and more interviewees might have given missing answers, in case they felt they were being asked too specific details.

The second hypothesis of our study is confirmed in its first part, namely that problematic gamblers have lower social stigma and more positive attitude towards gambling. In particular, social stigma of problematic gamblers with respect to lack of self-control, being irresponsible, being untrustworthy and having opinions that should not be taken seriously, is much lower than for non-problematic/non gamblers. Irresponsibility has been highlighted also by Horch and Hodgins (2015). Self-control is a feature that problematic gamblers believe to possess, despite the opposite evidence. The second part of the second hypothesis, namely that problematic gamblers have the lowest stigma and the most positive attitude, whilst non-gamblers who do not know problematic gamblers have the highest stigma and the least positive attitude towards gambling is confirmed, but differences are statistically significant only for the former group of people with respect to non-gamblers, regardless of knowing or not problematic gamblers. Research on other objects of stigma revealed that to know someone who has the features that are stigmatised (e.g., HIV) reduces stigma, but seems not to be confirmed by our findings. In our findings, only two items show significant differences between those who know and those who do not know problematic gamblers, namely: the one stating that gamblers are greedy and the one on the fact that gambling should be prohibited. These two facts lead us to say that a possible limitation of the tools employed for this research was that they were psychometrically calibrated so as to measure social and self-perceived stigma in a general population, and not to differentiate between those who know and those who do not know gamblers. We conjecture that a different formulation of the items of the questionnaire, which explicitly refer to known gamblers, may lead to different results and this remains an open issue for future studies.

Focusing on the subsample of problematic gamblers, which represent a percentage of 6.2% of the interviewees and are slightly less than expected if compared to other Italian studies (e.g., Picucci et al., 2013), we found out that social stigma and its two sub-dimensions of contempt and ostracism significantly predict self-perceived one. Namely, the more the social stigma, the more the self-perceived stigma. Luoma et al.’s (2007) interpretation that stigmatised people feel to be so not only on the basis of what they directly experience, but mostly on the basis of socially perceived stigmatisation, seems to be confirmed by our findings. Referring to the fourth hypothesis, we further provide evidence that the more severe the addiction, the higher the self-perceived stigma. This could have an effect on help-seeking, as in our study problematic gamblers who ask for help live with a significantly lower self-perceived stigma than those who do not ask for it. We thus confirm Hing et al.’s (2016) finding that lower self-stigma corresponds to higher request for help.

In the open answers concerning help-seeking, among both problematic gamblers and people who know at least one problematic gambler, the belief of being able to cope with the problem alone emerges, as in Hodgins and Cunnigham (2009), and Suurivali et al. (2009), but also new nuances are offered, such as: the will to dispose of one own’s money and the lack of awareness of having a disorder. Referring back to Goffman’s (1963) defense mechanisms, the belief of being able to cope with the problem alone can be linked to correcting strategies, but we can add new defence mechanisms, namely: the one of ignoring to have a problem and the one of pretending to be considered normal and able to use one own’s money.

Shame, which emerged in previous findings, is mentioned by a small but interesting percentage of people who know problematic gamblers.

With respect to Jones et al.’s (1984) six dimensions of stigma and possible consequences for not seeking for help, it seems that course does not emerge, namely it is not mentioned by the interviewees whether problematic gamblers can get out of the disorder or not as a reason for not searching for treatment. Noticeable features of gambling disorder, which emerged in Hing et al.’s (2016) study and open the possibility that significant others can prompt gamblers to search for help, are not mentioned as well in our sample.

## Conclusions

In our study, we confirmed that gambling-related social stigma is spread across socio-demographic variables. This does not allow us to suggest ways of targeting interventions to reduce stigma, but the relations between attitude and instruction may encourage us to suggest to consider educational interventions as viable ways to lower social stigma towards gambling, promoting a positive view of treatment. This is particularly relevant considering our finding that higher self-perceived stigma corresponds to more severe disorder and less request for help. Recalling the findings by Horch and Hodgins (2015), namely that positive attitude towards treatment is related to higher frequency of help-seeking, education can represent a way to affect attitude, thus helping reduce stigma as an indirect effect.

Being confirmed that problematic gamblers have less social stigma, we see as a future line of research a focus on non-problematic gamblers and non-gamblers as separate groups, and on a distinction between those who know problematic gamblers and those who do not, to investigate differences in social stigma among these different samples. It seems also necessary to reformulate the items of the GPSS and GESS, so as to allow a better focus on relatives and friends that can have problematic gambling behavior. This would allow us to develop an extension of Donaldson et al.’s (2015) tool, which will allow us to better understand social stigma with respect to how close are the stigmatising persons to the stigmatised ones.

## Footnotes

1 In our study, we consider the adjectives social and perceived as synonyms when referred to stigma, and experienced and self-perceived as synonyms as well.

2 In our research, the sample of problematic gamblers we refer to is the one identified by this test as people scoring 3 points or above. The other participants are considered non-gamblers or non-problematics gamblers and we have no means to further distinguish between these two categories.
